# Application and Surgical Technique of ACL Reconstruction Using Worldwide Registry Datasets: What Can We Extract?

**DOI:** 10.3390/jfmk7010002

**Published:** 2021-12-27

**Authors:** Ulrike Wittig, Georg Hauer, Ines Vielgut, Patrick Reinbacher, Andreas Leithner, Patrick Sadoghi

**Affiliations:** Department of Orthopaedics and Trauma, Medical University of Graz, 8036 Graz, Austria; ulrike.wittig@medunigraz.at (U.W.); georg.hauer@medunigraz.at (G.H.); ines.vielgut@medunigraz.at (I.V.); patrick.reinbacher@medunigraz.at (P.R.); andreas.leithner@medunigraz.at (A.L.)

**Keywords:** anterior cruciate ligament, ACL, ACL reconstruction, knee ligament register

## Abstract

Anterior cruciate ligament (ACL) injuries are among the most common knee injuries. The purpose of this study was to compare surgical reconstruction of the ACL between different countries and regions in order to describe differences regarding epidemiological data, reconstruction frequency, and graft choice. A systematic literature search was performed using the ACL study group website in order to identify the relevant knee ligament registers. Four national registries were included, comprising those from Sweden, the UK, New Zealand, and Norway. A large variation was found concerning the total number of primary ACL reconstructions with a reported range from 4.1 to 51.3 per 100,000 inhabitants. The country-specific delay between injury and reconstruction varied between an average of 6.0 months and 17.6 months. The leading sports activities resulting in ACL injury included soccer, alpine skiing, handball, rugby, and netball. Moreover, a strong variability in graft choice for primary reconstruction was found. The comparison of ACL registers revealed large differences, indicating different clinical implications regarding conservative or surgical therapy and choice of the preferable graft. ACL registers offer a real-world clinical perspective with the aim to improve quality and patient safety by investigating factors associated with subsequent surgical outcomes.

## 1. Introduction

Anterior cruciate ligament (ACL) injuries are among the most common knee injuries, with an incidence of 68.6 per 100,000 inhabitants [1]. Particularly in athletes, ACL rupture is reported as the knee ligament injury that most often requires surgical reconstruction [2]. Moreover, studies have shown a steady increase of ACL reconstructions in recent years [1,3]. Along with symptomatic knee instability, ACL injury involves an increased risk of meniscal and cartilage lesions and hence premature osteoarthritis (OA), regardless of treatment [4,5]. Incidence of knee OA is known to rise from 15% to 20% in ACL-deficient knee, representing a ten-fold increase. In other words, studies show that more than half of all patients suffering an ACL injury will develop symptomatic osteoarthritis in the following 10 to 20 years [6,7]. The primary aims of ACL reconstruction include restoration of normal knee kinematics, stabilization of the knee joint, and hence prevention of additional chondral and meniscal damage. Nevertheless, some studies have shown increased incidence of osteoarthritis even after surgical reconstruction of ACL [8,9].

ACL registers enable prospective surveillance of a selected patient population to gain long-term follow-up data of patients and thereby provide feedback to surgeons to improve patient safety and quality. Several ACL registers from all over the world have aimed to summarize country-specific patient characteristics, surgical techniques, graft choice, and revision rates after ACL reconstruction. The generalization of outcomes from single registries to other populations throughout the world still remains unclear, as patient- as well as surgeon-dependent factors varying between countries play a role. A cross-registry analysis gives the opportunity to evaluate surgery-related factors globally and enables interaction and share of knowledge between physicians. Furthermore, it yields the chance to improve patient outcomes after treatment of ACL injuries due to a large database summarizing different therapeutic strategies for ACL reconstruction and their respective outcomes [10].

The purpose of this study was to compare surgical reconstructions of the ACL between different countries and regions in order to describe differences regarding procedure types, including surgical technique and graft type, and frequency. Furthermore, epidemiological data and patient demographics were extracted from worldwide ACL registers. The authors hypothesized that ACL register analysis would reveal major differences between countries concerning the number of ACL reconstructions due to supposedly different approaches regarding conservative and surgical therapy, as well as regarding graft choice due to assumed country-specific different preferences.

## 2. Materials and Methods

### 2.1. Search Strategy

A systematic literature search was conducted using the ACL study group website in order to identify the relevant knee ligament registers [11]. In addition, a free-hand search using the search keywords “(ACL register) OR (knee ligament register)” was performed via Google. The final search date was 20 September 2021. This method is widely accepted and has been applied repeatedly in the field of orthopedics concerning the use of arthroplasty registers [12,13,14].

ACL registers that fulfilled the following inclusion criteria were considered for evaluation: (1) Reports had to be publicly available, (2) reports had to be written in English language, and (3) data had to be reported from the years 2019 or 2020 to retrieve recent data. Exclusion criteria included reports with updates older than 2019 as well as reports not available in the English language and registers not publicly available.

### 2.2. Study Selection and Outcomes

The relevant ACL registers were searched to find the annual report from 2020 or, if not available, from 2019. Data were extracted with respect to the number of performed ACL reconstructions, patients’ age distribution, patients’ activity in connection with ACL injury, time between injury and reconstruction, and procedure types and type of ACL graft chosen in primary reconstructions.

Eligibility of the registers was assessed independently and in duplicate and the assessments were crosschecked afterwards. Disagreement was resolved by discussion or, if necessary, by the decision of the senior author according to the Preferred Reporting Items for Systematic Review and Meta-Analysis (PRISMA) guidelines [15].

Three countries presented their data in the form of an annual report for each year separately, whereas the register from the United Kingdom (UK) reported summarized data from the year of establishment of their ACL register until the most recently documented time period. Registers used different classifications with respect to age distribution and time between injury and reconstruction and were pooled, if applicable.

### 2.3. Data Analysis

Methodologically, real life data and no “probabilities” were analyzed in this study. Hence, no *p* values could be calculated as previously published [16]. In this study, to compare all included registers, annual total number of primary ACL reconstructions per 100,000 inhabitants were calculated.

In general, due to limited data and the use of different reporting techniques, the present review focused on descriptive analysis of the results.

## 3. Results

The primary search yielded seven knee ligament registers. After applying the predefined inclusion and exclusion criteria, four registers (Sweden, Norway, the UK, and New Zealand) fulfilled the criteria and offered sufficient data. Hence, all four were included in the final analysis.

**Figure 1:**
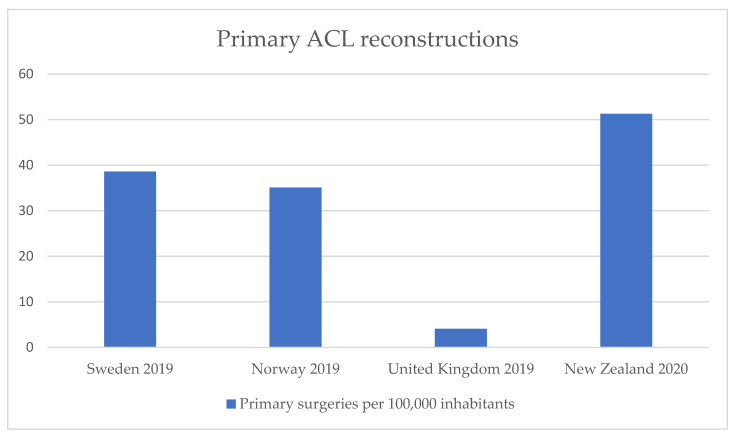
Reported number of annual primary anterior cruciate ligament (ACL) reconstructions per 100,000 inhabitants in different worldwide ACL registers.

A large variation was found concerning the total number of primary ACL reconstructions in 2019 with a reported range from 4.1 to 51.3 per 100,000 inhabitants, as depicted in Figure 1.

Patients’ mean age at primary surgery was reported in all registers but the Norwegian report. Mean age at surgery ranged from 28.0 to 29.2 years. The leading sports activities causing ACL injury mainly included soccer, alpine skiing, handball, rugby, and netball. A strong difference between the four countries was found concerning the time span between injury and primary reconstruction surgery. This variable was not reported in the Norwegian ACL register. The delay between injury and reconstruction varied between an average of 6.0 months in the UK, an average of 10.1 months in New Zealand, and an average of 17.6 months in Sweden. A summary of these variables is presented in Table 1.

**Figure 2:**
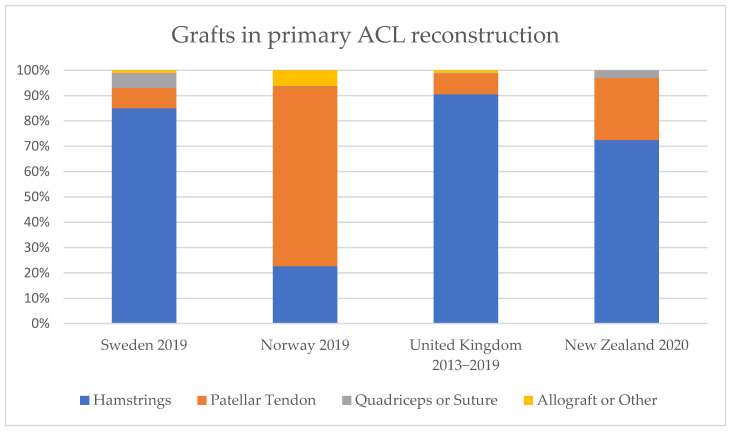
Percentage frequency of different grafts used in primary ACL reconstructions.

Moreover, there was a strong variability regarding graft choice for primary reconstruction between the different countries, as shown in Figure 2.

For example, hamstring tendons (HT) accounted for 90% of grafts in primary ACL reconstructions in the UK, 71% in New Zealand, and 85% in Sweden, but made up only 23% in Norway, respectively, where 71% of grafts in primary ACL reconstructions were patellar tendon grafts (PT). Quadriceps tendons and allografts were rarely used in all four countries, with percentages ranging from 0% to 2% of grafts in primary reconstructions.

## 4. Discussion

The purpose of this study was to compare surgical reconstructions of the ACL between different countries and regions in order to describe differences regarding procedure types and frequency, and to report clinical implications. The authors’ hypotheses regarding differences in frequency and graft choice were eventually accepted.

One of the most important findings of this study was a large variation concerning the annual number of primary ACL reconstructions ranging from 4.1 to 51.3 per 100,000 inhabitants. In an Australian population-based study, this figure was slightly higher with an average of 52.0 procedures per 100,000 inhabitants [17]. Population-based studies from other countries or regions presented lower figures, including incidences from 28.7 to 35.2 per 100,000 inhabitants in population-based data from the United States, 32.0 per 100,000 citizens in Germany, and 38.0 per 100,000 inhabitants in Denmark, respectively [18,19,20,21]. Publications of pooled data within countries that have their own ACL registers showed quite similar results to registry data for Norway and Sweden, with incidences of 34.0 and 32.0 per 100,000 inhabitants, respectively [22,23], except for differences regarding New Zealand. A pooled analysis by Gianotti et al. in 2009 showed an incidence of 37.8 per 100,000 inhabitants, while the register from 2020 presented an incidence of 51.3 per 100,000 [2]. This might be explained by the significant increase of ACL reconstructions in New Zealand in recent years. In general, differences in reconstructions per 100,000 inhabitants could be explained by different sport habits but also health economic factors, for example the insurance system and cost coverage for surgery. Moreover, these rather large country-specific differences might indicate different tendencies such as whether to aim for a conservative or surgical treatment approach primarily due to varying experiences and country-specific knowledge from the previous decades.

A strong difference was found regarding the delay between injury and reconstruction, which was not reported in the Norwegian register. That timespan varied between an average of 6.0 months in the UK, an average of 10.1 months in New Zealand, and an average of 17.6 months in Sweden. In 2009, Granan et al. reported a median time (in months) from injury to surgery of 7 months (range 0–482) in Norway, 9 months (range 0–371) in Denmark, and 10 months (range 0–527) in Sweden [23]. In a private institutional ACL register from a private hospital in Brazil, the median time from injury to surgery only amounted to 44 days [24]. This discrepancy might be explained by different national traditions concerning treatment of ACL injury and differing favoritism of trying a conservative treatment approach by muscle strengthening and only switching to surgical treatment if stabilization of the knee joint cannot be achieved by conservative therapy. In addition, differences in national health care systems and different requirements for cost coverage by insurances could play a role.

The leading sports activities causing ACL injury included soccer, alpine skiing, handball, rugby, and netball in all four registers. This was confirmed in an Australian population-based study, where skiing, soccer, football, rugby, and netball showed the highest incidences of ACL reconstructions per 100,000 participants [17]. In Denmark, the foremost sports activities causing ACL injury were soccer, handball, alpine sports, and football [18]. Additionally, even slight differences in country-specific sports preferences might have an impact on the frequency of ACL reconstructions in the respective countries, for example, if some high-risk sports are performed more commonly in one country compared to the other.

Furthermore, significant differences with respect to graft choice in primary reconstructions were observed. Hamstring tendons accounted for 90.0% of grafts in primary ACL reconstructions in the UK, 71.0% in New Zealand, and 85.0% in Sweden, but made up only 22.7% in Norway, respectively, where 71.0% of primary ACL grafts were patellar tendon grafts. Quadriceps tendons and allografts were barely used across all four countries. In an analysis from a Brazilian private institutional register, hamstring tendons were the most commonly used grafts with a share of 70.2%, followed by bone-tendon-bone patellar grafts with a share of 28.8%. Quadriceps tendons were only used in 0.8% of cases. This is in line with our findings regarding all countries except for Norway, as depicted in Figure 2 [24]. Moreover, our findings were also similar to those from Denmark, where 70.8% of primary ACL reconstructions were performed using hamstring tendons and 22.0% were done using patellar tendon grafts, respectively. In a systematic review, including eight national surveys from Europe, North or Latin America, and Asia, the hamstring tendon graft was reported as the preferred graft, with a share of 45.0% to 89.0% in the surveyed population, followed by patellar tendon graft, with a share of 2.0% to 41.0%, and allograft, with a share of 2.0% to 17.0% [25]. Variations in graft choice may be explained by several factors, such as patient demographics (country-specific sports habits differing in risk of causing injury) and national conditions, such as differences in healthcare systems (insurance status), number and availability of performing surgeons, medical facilities, and surgeon-dependent factors, such as definition of indications, education, tradition in graft choice, and experience [8,26,27,28,29,30,31,32,33,34,35].

Worldwide knee ligament registers are essential to compare country-specific differences and share experience between countries, especially concerning the early identification of inferior clinical outcomes associated with a particular graft or surgical technique and to determine factors for the optimization of patient care. Thus, the comparison of ACL registers entails implications for daily clinical practice. In addition, ACL registers, as well as knee ligament and arthroplasty registers in general, represent important tools to support the health care system, as ACL reconstruction contributes to health care costs significantly. Hence, collecting and sharing data in ACL registers can help to reduce expenses regarding treatment and postoperative rehabilitation. Moreover, knee ligament registers provide feedback to surgeons as well as patients [17].

### Limitations

There are several limitations to this present study. First, outcomes of patients with a conservative treatment approach for their ACL rupture were not tracked, and therefore, this report is limited to patients with a reconstructed ACL. Second, this study is of a descriptive nature and conclusions between certain risk factors and consequential outcomes after surgical reconstruction cannot be drawn. Third, reported findings from the UK registry may be contorted as this register was established more recently compared to the others and thus includes a smaller number of reconstructions. Furthermore, registries were not written according to standardized structures hence, data were sometimes reported in different manners, and comparisons, including graphical ones, could not be performed. Moreover, some exact data were missing in some reports, for instance patients’ age or the percentage frequencies regarding activities in connection with ACL injuries in the Norwegian report. Additionally, in the Swedish registry, percentage frequencies regarding activities associated with ACL injuries were reported separately by gender.

## 5. Conclusions

A comparison of worldwide ACL registers revealed large differences regarding the annual number of primary ACL reconstructions per inhabitant and concerning ACL procedure types, especially different preferences in graft choice, indicating different clinical implications regarding conservative or surgical therapy and choice of preferable graft. ACL registers offer a real-world clinical perspective with the aim to improve quality and patient safety by investigating factors associated with subsequent surgical outcomes.

## Figures and Tables

**Table 1:** Country-specific differences regarding activities associated with ACL injury, patients’ mean age at surgery, and delay to surgery.

.	Activities Associated with ACL Injury	Mean Age at Surgery	Delay to Surgery
Sweden 2019	Women:	28.0 years	17.6 months
	1. Skiing 27%		
	2. Soccer 26%		
	3. Handball 8%		
	Men:		
	1. Soccer 50%		
	2. Skiing 10%		
	3. Floorball 8%		
Norway 2019	1. Soccer °	n.e.*	n.e.*
	2. Skiing °		
	3. Handball °		
UK 2019	1. Soccer 48%	29.0 years	6.0 months
	2. Rugby 12%		
	3. Skiing 12%		
New Zealand 2020	1. Rugby 28%	29.2 years	10.1 months
	2. Football 15%		
	3. Netball 14%		

* n.e. = not extractable from registry, ° no percentage frequencies for activities associated with ACL injury.

## Data Availability

The datasets used and/or analyzed during the current study are available from the first author on reasonable request.
